# Dual-targeting biomimetic delivery for anti-glioma activity *via* remodeling the tumor microenvironment and directing macrophage-mediated immunotherapy†
†Electronic supplementary information (ESI) available: Tables S1–S3 and Fig. S1–S6. See DOI: 10.1039/c7sc04853j


**DOI:** 10.1039/c7sc04853j

**Published:** 2018-01-31

**Authors:** Pengfei Zhao, Yonghui Wang, Xuejia Kang, Aihua Wu, Weimin Yin, Yisi Tang, Jinyu Wang, Meng Zhang, Yifei Duan, Yongzhuo Huang

**Affiliations:** a Shanghai Institute of Materia Medica , Chinese Academy of Sciences , 501 Haike Rd , Shanghai 201203 , China . Email: yzhuang@simm.ac.cn ; Fax: +86-21-2023-1981 ; Tel: +86-21-2023-1981; b Zhejiang Academy of Medical Science , 182 Tianmushan Rd , Hangzhou 310013 , China; c Nanchang University College of Pharmacy , 461 Bayi Rd , Nanchang 330006 , China; d Guangzhou University of Chinese Medicine Tropical Medicine Institute , 12 Jichang Rd , Guangzhou 501450 , China

## Abstract

Tumor-associated macrophages (TAMs) are the major components in the tumor microenvironment (TME). The polarization from the protumor M2 (TAM2) to antitumor M1 (TAM1) phenotype can not only lift the immunosuppressive constraints and elicit cytotoxic T-cell immunity but also augment the chemotherapy efficacy. However, the treatment feasibility by TAM modulation in brain tumors and the mechanisms remained unknown. A dual-targeting biomimetic codelivery and treatment strategy was developed for anti-glioma activity. We demonstrated that the albumin nanoparticles modified with dual ligands, a transferrin receptor (TfR)-binding peptide T12 and mannose, efficiently passed through the BBB *via* the nutrient transporters (*i.e.*, TfR and the albumin-binding receptor SPARC) that were both overexpressed in the BBB and glioma cells, thus achieving biomimetic delivery to glioma. Importantly, after penetrating the BBB, this system can take advantage of the overexpression of the SPARC and mannose receptors on TAM2, thus also targeting the protumor TAM2. With the codelivery disulfiram/copper complex and regorafenib, the system efficiently inhibited the glioma cell proliferation and successfully “re-educated” the protumor TAM2 towards antitumor TAM1. The treatment efficacy was examined in the glioma-bearing nude mice and immunocompetent mice. It showed this system yielded an enhanced treatment outcome, owing to the synergistic combination of chemotherapy and macrophage-directed immunotherapy. The importance of this delivery and therapeutic strategy was to remodel the immune microenvironment and reprogram TAM and trigger macrophage-directed anti-glioma immunotherapy *via* the interplay of the TAM, Treg, and CD8^+^ T cells and the effector cytokines. The albumin-based biomimetic brain delivery also provides a promising method for the pharmacotherapy of brain diseases.

## Abstract 2

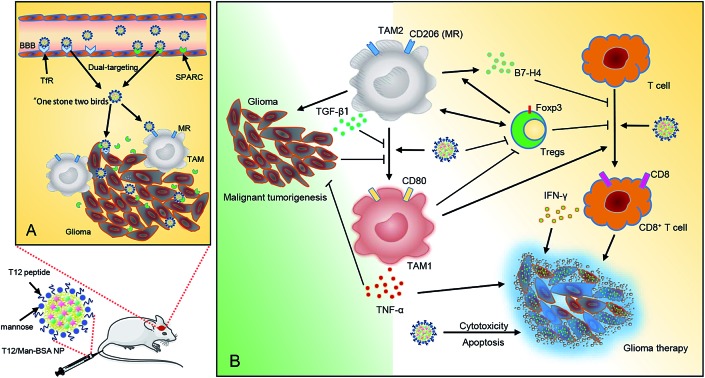
A dual-targeting biomimetic codelivery and treatment strategy was developed for anti-glioma activity.

## Introduction

Cumulative evidence indicates that clinical responses to chemotherapy can be improved if the immune cells are activated and the suppressive restraints lifted.^1^ The important role of the tumor immune microenvironment in controlling tumor progression has been well established. In cancer, immune cells can be a double-edged sword, with the ability to either arrest or support malignancy.^2^ A tumor-associated macrophage (TAM) is a major population of immune cells that affects tumor development.^3^ TAMs are highly plastic and, during tumor progression, TAMs can polarize to two major phenotypes: the antitumor M1 (TAM1) and the protumor M2 (TAM2). TAM1 is characterized by the high expression of pro-inflammatory cytokines like TNF-α, while TAM2 has high expression of anti-inflammatory cytokines like TGF-β. TAM2 serves as an important driving factor in immunosuppressive TME and, for instance, the secreted TGF-β induces regulatory T cells (Treg) and inhibits CD8^+^ T-cell responses to kill the cancer cells.^4^ The population of TAM1 or TAM2 can be used as a prognostic factor in cancer therapy.^5^

Malignant glioma is one of the most aggressive cancers and, due to the limit of the blood-brain barrier (BBB), very few chemo drugs yield productive treatment outcomes. Temozolomide (TMZ) is the first-line therapy for glioma. However, at least 50% of TMZ treated patients do not respond to TMZ^6^ and more than 90% of recurrent gliomas show no response to repeated challenges with TMZ.^7^ Therefore, there is an urgent need to develop a novel treatment. A traditional perception of “immune privilege” in the central nervous system has been challenged, and two recent important discoveries have revealed the existence of a lymphatic system both in the murine and human brain.^8^,^9^ Yet, the feasibility of brain cancer immunotherapy still remains largely unknown due to the impermeable BBB, which rejects most therapeutics, including antibodies. Recently, the first large randomized clinical trial (CheckMate 143, NCT 02017717) of PD-1 immunotherapy failed to prolong overall survival of patients with recurrent glioblastoma, and therefore did not reach its primary endpoint.^10^

Immune suppression has been found to be common in glioma,^11^,^12^ in which a surprisingly high portion of the cells, as many as 30–50%, are monocytes (macrophages and microglia).^13^ Remodeling the tumor immune microenvironment *via* modulation of the TAM polarization has been emerging as a new therapeutic target.^14^ Suppressing TAM2 and enhancing polarization towards TAM1 not only activates the cytotoxic T cells but also directly induces the secretion of antitumor cytokines from TAM1. However, there is little known about the feasibility of the combination of chemotherapy and TAM-based immunotherapy, specifically, for anti-glioma activity. Therefore, to explicate the effects of targeting and modulating TAM in a glioma microenvironment would be of great importance to provide critical understanding for developing novel anti-glioma strategies.

Here, we presented an albumin-based biomimetic delivery system for codelivery of the disulfiram/copper complex (DSF/Cu) and the macrophage modulator regorafenib (Rego) *via* a so-called “two-birds-one-stone” strategy for targeting both glioma cells and TAM2. Specifically, the albumin nanoparticles can target the albumin-binding proteins (*e.g.*, SPARC, secreted protein acidic and rich in cysteine), which are overexpressed in the tumor cells (*e.g.*, glioma) and tumor vessel endothelial cells associated with neoplasia for facilitating the uptake of albumin as a source of energy and amino acids.^15^,^16^ More interestingly, we recently discovered that SPARC was also highly expressed on TAM2, which becomes a potential therapeutic target of an albumin nanoparticulate system.^17^ In addition, we used the transferrin receptor (TfR)-binding peptide T12 (THRPPMWSPVWP) and mannose to modify the albumin nanoparticles to further enhance the glioma- and TAM2-targeting efficiency. The T12 peptide can promote BBB penetration and glioma cellular uptake, while the mannose ligand can specifically bind to the mannose receptors (MRs) on TAM2. Of note, an advantage of the T12 peptide is that it binds a site on TfRs distinct from the one to which transferrin binds, thus avoiding potential competition of endogenous transferrin for receptor binding.^18^ Therefore, by targeting these nutrient transporters (*i.e.*, TfR and SPARC), the functional albumin NPs could achieve biomimetic codelivery to the glioma.

However, both DSF/Cu and Rego are poorly BBB-permeable, and thus they have limited therapeutic value in anti-glioma activity. A benefit of codelivery design is that the combo drugs could display relatively identical pharmacokinetics profiles in glioma delivery, thus yielding a stochastic distribution to cells.^19^

## Results

### Characterization, cellular uptake, and tumor spheroid penetration of the NPs

**Fig. 1:**
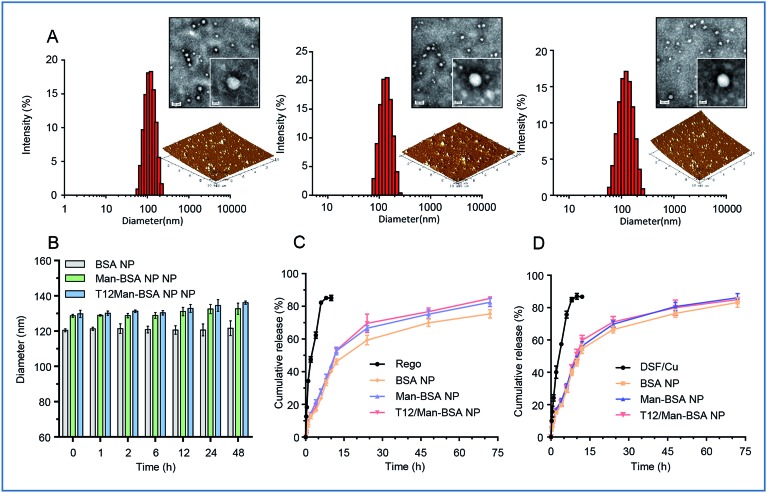
(A) The size distribution, TEM, and AFM images of the NPs (from left to right: BSA NPs, Man-BSA NPs, T12/Man-BSA NPs). (B) The stability of the NPs in PBS containing 10% FBS. (C) The *in vitro* release of Rego from the albumin NPs. (D) The *in vitro* release of DSF/Cu from the albumin NPs.

The albumin NPs were fabricated by a green method *via* urea/NaBH_4_ albumin denaturation as described in our previous work.^16^ All the drug-loaded albumin NPs were less than 135 nm with a narrow particle size distribution and negative *ζ*-potential (Table S1, ESI†). The transmission electron microscopy (TEM) photographs and atomic force microscopy (AFM) photographs showed that the NPs were spherical in shape and uniform in size (Fig. 1A). The NPs exhibited good stability and a sustained-release pattern (Fig. 1B–D).

**Fig. 2:**
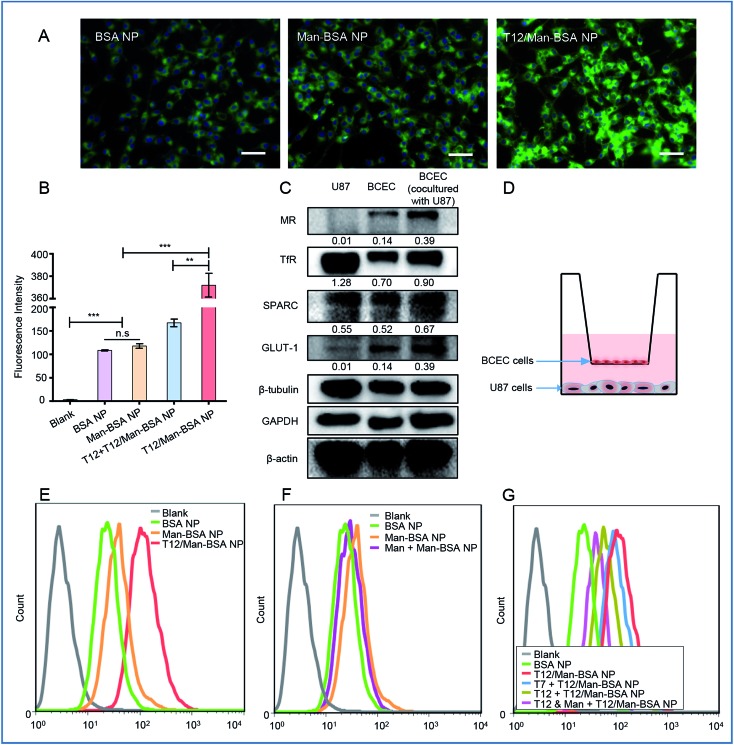
The cellular uptake studies. (A) The fluorescence images of the uptake of the NPs in the U87 cells (scale bar: 50 μm). (B) The cellular uptake efficiency in the U87 cells. (C) The expression of the transporters of MRs, TfRs, SPARC, and GLUT-1 in the U87 and BECE cells. (D) A schematic diagram of the Transwell co-culture model. (E) The transwell uptake efficiency of the various NPs in the lower chamber U87 cells. (F) The inhibition of the Transwell uptake of the Man-BSA NPs in the U87 cells in the lower chamber with a BCEC monolayer pretreated with mannose. (G) The inhibition of the Transwell uptake of the T12-BSA/Man-BSA NPs in the U87 cells with a BCEC monolayer pretreated with mannose and T7/T12 peptide.

The T12/Man-BSA NPs displayed 3-fold higher uptake efficiency in the human glioma U87 cells than the non-modified BSA NPs and Man-BSA NPs (Fig. 2A, B and S3C†). Because the U87 cells expressed a very low level of MRs and GLUT-1 (Fig. 2C), there was no major difference between the groups of the Man-BSA NPs and BSA NPs. However, there was an approximate two-fold decrease in the uptake efficiency of the T12/Man-BSA NPs if the cells were pretreated with the free T12 peptide (Fig. 2B and S3C†), demonstrating the importance of the TfR-mediated mechanism for the uptake of the T12/Man-BSA NPs.

A monolayer culture in a Transwell with brain capillary endothelial cells (BCECs) in an upper chamber and U87 cells in a lower chamber is a commonly used *in vitro* model for brain delivery studies.^20^ Both TfRs and SPARC were overexpressed in the BCEC and U87 cells and, interestingly, their expression in the BCECs was further upregulated by co-culture with the U87 cells (Fig. 2C). The Transwell uptake of the T12/Man-BSA NPs by the U87 cells was about 2-fold higher than with the BSA NP (Fig. 2E), demonstrating the ability of the T12 peptide to further enhance the penetration through the BCEC monolayer and the uptake by the U87 cells. The uptake efficiency of the Man-BSA NP was higher than the BSA NP (Fig. 2F), which could be related to the BCEC cells expressing glucose transporters 1 (GLUT-1) and MRs (Fig. 2C). GLUT-1 can mediate the delivery across the BBB for those substances with similar structures of glucose, including mannose and glucose analogs.^21^,^22^ As evidence, with the BCEC pretreated with mannose, the uptake of the Man-BSA NP in the U87 cells was reduced, exhibiting an efficiency similar to the BSA NP (Fig. 2F). Moreover, GLUT-1 colocalized with the distribution of the Man-BSA NPs in the tumor tissues (Fig. S5†), indicating that GLUT-1 mediated delivery for tumor targeting was an important mechanism. If the BCECs were pretreated with the T12 peptide, the U87 cell uptake efficiency of the T12/Man-BSA NPs was significantly reduced (Fig. 2G). However, the pretreatment of the T7 peptide (sequence: HAIYPRH), a commonly used TfR-targeting ligand for brain delivery,^23^,^24^ did not effectively block the transcellular transport, owing to the much higher TfR affinity of T12 than that of T7.^18^ As expected, the pretreatment of both T12 and mannose could further decrease the penetration of the T12/Man-BSA NPs.

**Fig. 3:**
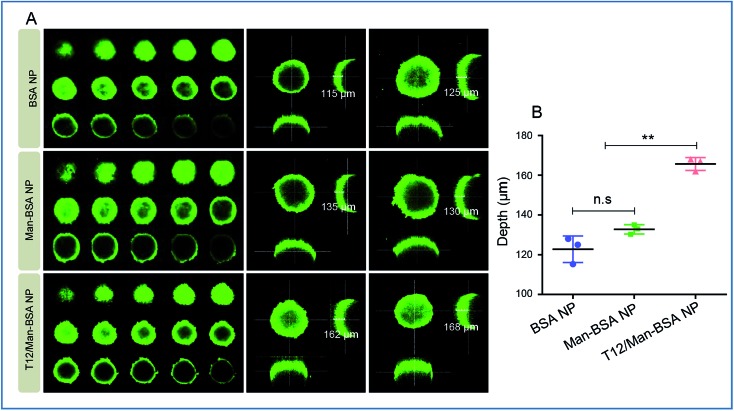
The intra-spheroid penetration studies. (A) The penetration into the U87 spheroids of the NPs. (B) The quantitative analysis of the penetration depth in the U87 spheroids (confocal *Z*-axis continuous top-down scanning layers, 20 μm for each depth level).

Due to the high interstitial fluid pressure, poor drug intratumoral infiltration is a daunting obstacle.^25^ In the cultured U87 spheroids, the T12/Man-BSA NPs showed a much deeper infiltration than the other two NPs (Fig. 3). We postulated that the T12/Man-BSA NPs was recruited and bound to the tumor spheroids by the overexpression of TfRs and SPARC, and the dual-receptor synergistic effect (SPARC and TfRs) promoting the diffusion into the spheroids.

### Cytotoxicity test and TAM repolarization

**Fig. 4:**
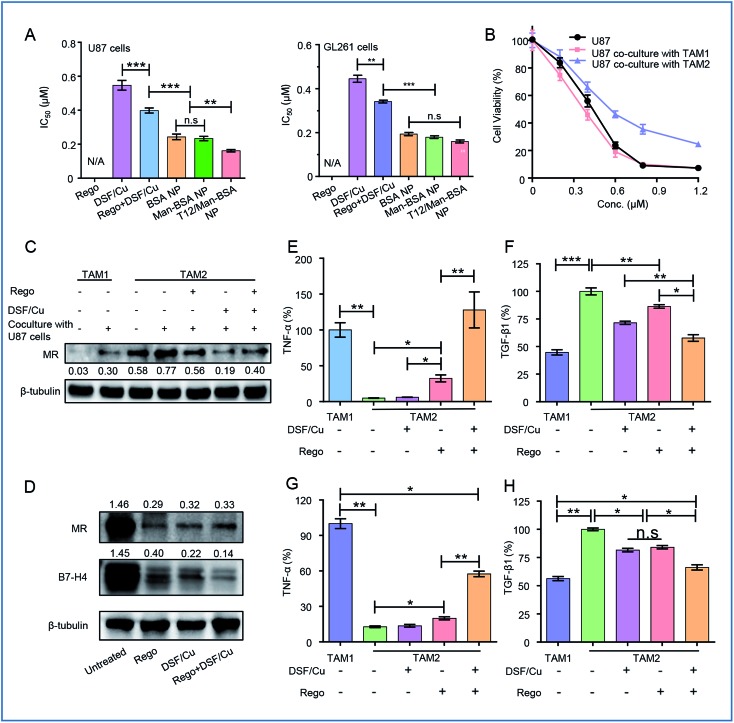
(A) The cytotoxicity test (IC_50_) in the U87 and GL261 cells. (B) The cytotoxicity test in the U87 cells cultured with M1-CM or M2-CM. (C) The expression of MRs in TAM1 and TAM2 and the polarization modulation of the drugs. (D) The re-education of TAM2 treated with drugs and the expression of MRs and B7-H4. The ELISA analysis of the expression of TNF-α (E) and TGF-β1 (F) in TAM1 and TAM2 after drug treatment. The ELISA analysis of the expression of TNF-α (G) and TGF-β1 (H) in TAM1 and TAM2 cocultured with U87 cells after treatment.

The synergistic effect of DSF/Cu and Rego was examined. The combination therapy (DSF/Cu and Rego) effectively inhibited the growth of the glioma cells, with a lower IC_50_ and higher apoptosis rate compared to the single use of DSF/Cu in the U87 cells (Fig. 4A, S1A and B†). There was also a similar trend observed in the GL261 cells (Fig. 4A, S1C and D†). The antitumor activity was further enhanced by using dual drug-loaded NPs. The T12/Man-BSA NPs displayed the strongest cell growth inhibition, with an IC_50_ of about 0.16 μM in the U87 cells, and 0.15 μM in the GL261 cells (Fig. 4A). But the Man-BSA NP and BSA NP showed similar cytotoxicities because of the lack of MR expression on both glioma cells. Accordingly, the T12/Man-BSA NP group displayed the highest apoptotic rate of 96.1% (early and late apoptosis) in the U87 cells (Fig S1B†) and 82.3% in the GL261 cells (Fig S1D†), whereas there were 67.2% in the U87 cells and 60.2% in the GL261 cells for the free combo drugs.

The most important feature of this two-birds-one-stone strategy was the simultaneous action on both the cancer cells and TAM—a major component of TME. During tumorigenesis, the recruited macrophages (MΦ) are gradually transformed into the M2 phenotype to promote tumor progression.^26^,^27^ However, the polarization of the TAM is reversible and appropriate interventions can “re-educate” the pro-tumor TAM2 into the antitumor TAM1.^28^–^30^ The effect of the NPs on the TAM was examined in the mouse bone marrow-derived macrophage (BMM)-differentiated TAM model. The cellular uptake of the NPs in the TAM2 cells was investigated, and the efficiency of both the Man-BSA NP and T12/Man-BSA NP was higher than the BSA NP (Fig. S3A and B†). It is suggested that the overexpressed MRs of TAM2 promoted the cellular uptake. Simultaneously, the supernatants from the TAM1 or TAM2 culture medium were collected (termed M1-CM and M2-CM) and added to the U87 cells. The IC_50_ of the M2-CM co-cultured U87 cells (DSF/Cu, 0.58 μM) was obviously higher than that of the co-cultured M1-CM (0.34 μM) and the untreated control (0.41 μM) (Fig. 4B). This confirmed the antitumor effect of TAM1 and the induction of the drug resistance of TAM2.

The TAM modulation effect of Rego has been reported.^31^ The combination of DSF/Cu and Rego was found to have a synergistic effect on the TAM polarization. With treatment of DSF/Cu and/or Rego, the IL-4-induced TAM2 was able to be “re-educated” towards TAM1 (Fig. 4C and D).

There is a complicated crosstalk network among various kinds of cells in TME (*e.g.*, TAM and the cancer cells). By exposure to the U87 cell culture medium, TAM1 was induced towards the protumoral TAM2 polarization, characterized by the upregulated MR expression (Fig. 4C). We also investigated the modulation effect of the drugs on the TAM2 exposed to the U87 cell medium to mimic TME. The drug treatment successfully “re-educated” TAM2 toward the antitumoral TAM1 (Fig. 4C).

The modulation of the TAM was also examined by measuring the cytokine levels. TAM2 treated with DSF/Cu and Rego showed up-regulation of the antitumor TNF-α and down-regulation of the pro-tumor TGF-β1 (Fig. 4E and F), demonstrating the re-polarization of the phenotype towards M1. The combo drugs (Rego + DSF/Cu) exhibited a better modulation effect than the single use of each drug. The TAM re-polarization effect of Rego + DSF/Cu was further confirmed in the TAM/U87 cocultures (Fig. 4G and H). The ROS production of DSF/Cu could be a mechanism for yielding the synergistic effect on TAM re-polarization.

### Biodistribution and *in vivo* imaging

**Fig. 5:**
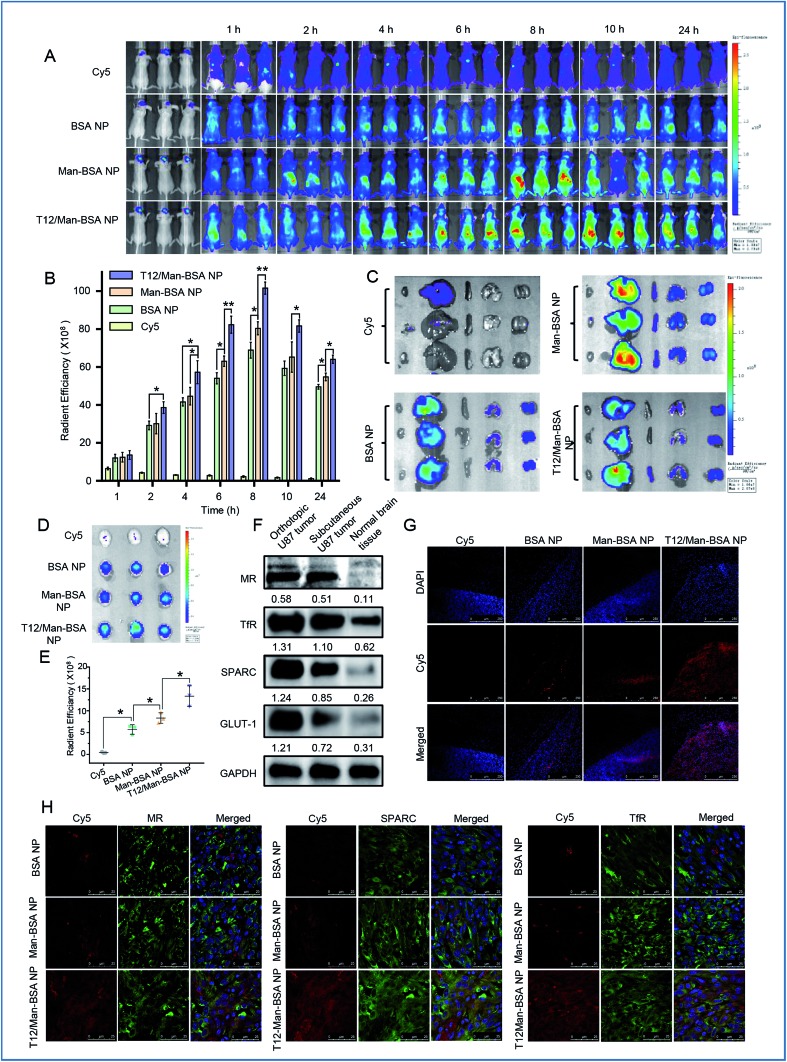
The *in vivo* imaging of the NP biodistribution in the mice bearing U87 orthotopic tumors. (A) The whole body imaging. (B) The *in vivo* radiant efficiency of the brain sites. (C) The imaging of the dissected major organs from the mice. (D) The imaging of the brains dissected from the mice. (E) The *ex vivo* radiant efficiency of the brains. (F) The expression of the nutrient transporters in glioma. (G) The intraglioma penetration of the Cy5-labeled NPs (red). (H) The immunofluorescence colocalization of the Cy5-labeled NPs and SPARC, TfRs, or MRs.

The brain accumulation of the T12/Man-BSA NPs reached the maximum at about 8 h after i.v. injection in an orthotopic U87 glioma Balb/c nude mouse model (Fig. 5A and B), with better glioma-targeting effect than the other two NPs. *Ex vivo* imaging showed higher brain accumulation of the T12/Man-BSA NPs (Fig. 5D and E), but lower liver disposition (Fig. 5C), compared to that of the BSA NPs and Man-BSA NPs.

The overexpression of the transporters, such as SPARC, TfRs, and GLUT-1, was demonstrated in both the orthotopic and subcutaneous U87 glioma models (Fig. 5F). Due to the abundancy of TAM2 in the tumor, MRs were also highly expressed in the glioma tissues. Therefore, the glioma-targeting delivery was associated with these transporters. In accordance with the results of the tumor spheroid penetration study, the T12/Man-BSA NPs maximally enhanced tumor penetration compared to the other two NPs and showed widespread distribution across the tumor region (Fig. 5G). This again demonstrated the glioma-targeting effect and ability to overcome the intratumoral heterogeneity barrier.

The importance of the transporters (*e.g.*, TfRs) was already revealed in the Transwell studies. To further investigate the active targeting delivery mechanism, the colocalization of the NPs and the transporters in the glioma tissue slices were observed using CSLM. As shown in Fig. 5H, all the albumin NPs showed colocalization with SPARC, indicating that the SPARC-mediated pathway was involved in the glioma-targeting delivery. To further confirm this pathway, a p38-MAPK inhibitor SB203580 was taken as the SPARC inhibitor.^32^,^33^ With pretreatment of SB203580, the U87 cell uptake efficiency of the BSA NPs was reduced, in accordance with the down-regulation of the SPARC expression (Fig. S3D and E†). This demonstrated that SPARC plays a key role in the tumor-targeted delivery of the albumin NPs. In addition, the T12/Man-BSA NPs were also colocalized with TfRs and MRs (Fig. 5H) and involvement of the TfR- and SPARC-pathways in BBB penetration and glioma-targeting delivery is suggested, along with the effect of the MRs on the targeting TAM2. In contrast, the Man-BSA NPs colocalized with MRs but not TfRs.

We have previously reported a glioma targeted cell-penetrating peptide-modified albumin NP (termed L-BSA NP).^16^ It would be an interesting observation to compare the *in vivo* fate of the L-BSA NP and T12/Man-BSA NP, and thus a pharmacokinetics study in rats was performed. The mean retention time (MRT) of the T12/Man-BSA NP was prolonged compared to the L-BSA NP (7.5 h *vs.* 6.4 h) and the AUC was larger (260.0 *vs.* 209.7 μg L^–1^ h^–1^) (Fig. S4A†). As expected, the bio-distribution of the L-BSA NP in the major organs was significantly higher than the T12/Man-BSA NP (Fig. S4B and C†). This may be related to the relatively wide penetration nature of the cell-penetrating peptides. Furthermore, Fig. 5E shows the enhanced brain targeting coefficient, *i.e.*, 2.34 for the T12/Man-BSA NP/BSA NP, compared with the value of 2.13 reported previously for the L-BSA NP/BSA NP.^16^

### *In vivo* therapy and MΦ-mediated immune responses

**Fig. 6:**
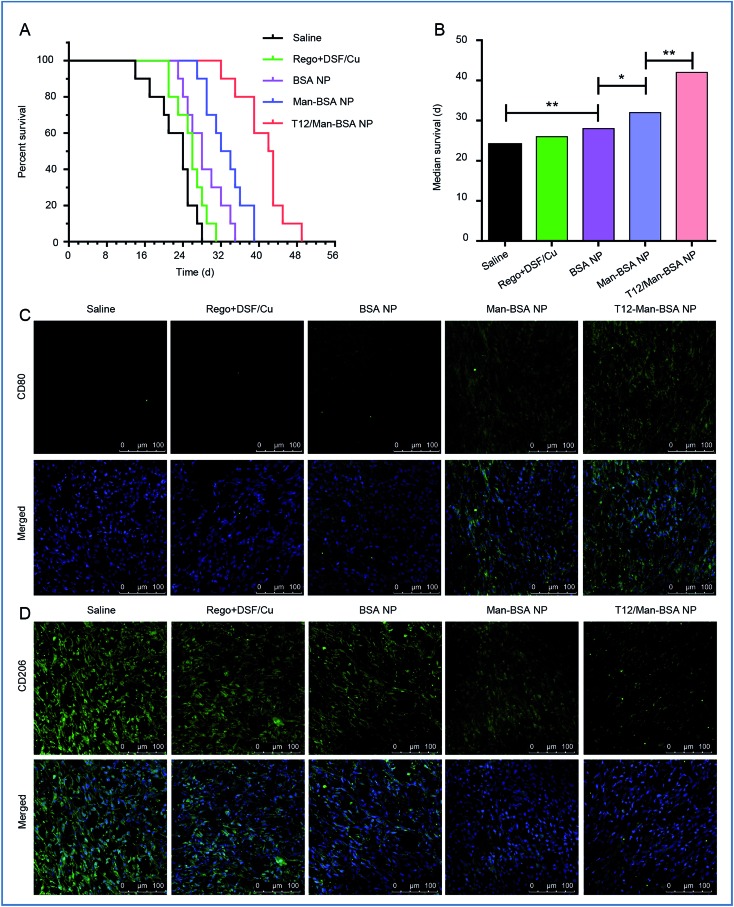
The treatment efficacy on the Balbc/nude mice bearing U87 orthotopic glioma. (A) The survival curve. (B) The median survival and statistical analysis of each treatment group. The expression of CD206 (M2 macrophage) (C) and CD80 (M1 macrophage) (D) in U87 orthotopic glioma of Balbc/nude mice after treatment.

The *in vivo* anti-glioma efficacy was investigated using transplanted orthotopic U87 glioma-bearing nude mice. The group given the T12/Man-BSA NPs displayed the longest survival time, with a median survival time of 42 days, compared to 32 days for the Man-BSA NP group and 28 days for the BSA NP group (Fig. 6A and B, Table S2†). The results demonstrated the important roles of the dual-targeting delivery in improving the anti-glioma therapy.

To verify the major mechanism was TAM2 “re-education” or blood recruitment, the BMMs (M1 polarization) were stained with CFDA SE and intravenously injected into the orthotopic U87 glioma-bearing mice. Fig. S6† shows that the labeled MΦ had insignificant intratumoral accumulation (with little green fluorescence). It is indicated that TAM2 “re-education” in TME could be the main mechanism but not blood recruitment. This was consistent with a previous report that regorafenib reduced the infiltrating macrophages but modulated TAM re-polarization.^31^

The therapeutic effect on TAMs was also investigated. It was found that the population of TAM1 (with marker CD80) was increased while that of TAM2 (with the marker CD206) was reduced (Fig. 6C and D) after treatment with the T12/Man-BSA NPs. This further supported the *in vitro* observation that the modulation of TAM polarization was an important therapeutic mechanism. Our results demonstrated the feasibility of directly “re-educating” TAM2 towards TAM1 in the glioma microenvironment using the T12/Man-BSA NPs, as well as the success of the “two-birds-one-stone” therapy strategy.

**Fig. 7:**
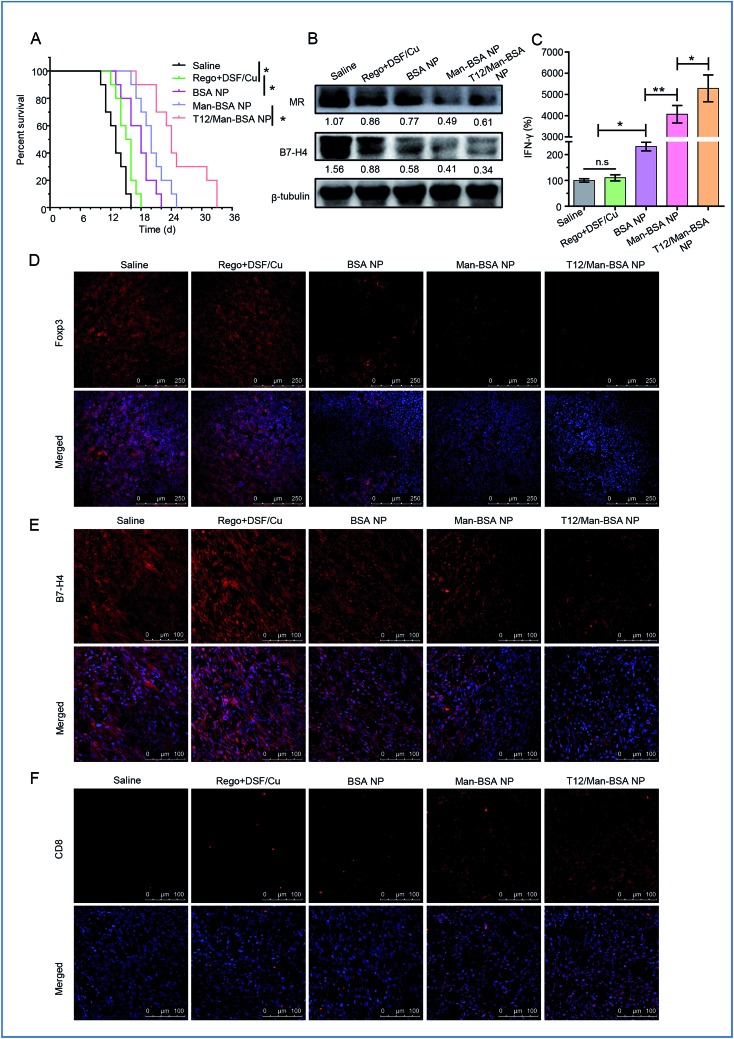
The treatment efficacy on the C57BL/6 mice bearing GL261 orthotopic glioma. (A) The survival curve. (B) The expression of the MRs and B7-H4 in glioma after treatment. (C) The IFN-γ level in the glioma after treatment. The population of Foxp3^+^ Treg (D), B7-H4^+^ TAM (E), and CD8^+^ T cells (F) in glioma after treatment.

The antitumor effect of TAM1 involves various mechanisms, such as the release of pro-inflammatory cytokines (*e.g.*, TNF-α) and the activation of cytotoxic T lymphocytes (CTL), and the importance of macrophage-directed immunotherapy has been noted.^34^ The anti-glioma efficacy was further investigated using a murine GL261 glioma-bearing immunocompetent C57BL/6 mouse model. The T12/Man-BSA NP group showed the best treatment outcomes, with a median survival time of 24 days, compared to 20 days for the Man-BSA NP group and 18 days for the BSA NP group (Fig. 7A, Table S3†). It should be mentioned that the murine GL261 orthotopic glioma model was highly aggressive, with rapid tumor progression. After treatment with various NPs, the MR expression was down-regulated, suggesting suppression of the TAM2 polarization in the glioma (Fig. 7B).

Recently, the mechanistic interplay between the TAMs and T cells has attracted attention and the modulation of TAMs can be a potential avenue for boosting the efficacy of immunotherapy.^35^,^36^ For example, TAM2 secretes TGF-β and induces regulatory T cells (Treg), and the increased proportion of Treg leads to the inhibition of the CD8^+^ T-cell responses.^4^ Foxp3 is a key transcriptional regulator molecule that regulates the differentiation and functions of Treg, thus serving as a marker for Treg activity.^37^ Our data revealed that the Treg was suppressed after treatment with the T12/Man-BSA NPs (Fig. 7D). In addition, there is a mechanistic interaction between Treg and MΦ. Treg can induce the expression of B7-H4, a B7 family member protein, on MΦ and convey the suppressive signal to inhibit T-cell immunity.^38^*Via* western blotting and immunofluorescent staining observation, B7-H4 was greatly down-regulated in the glioma tissues from the mice receiving the T12/Man-BSA NPs (Fig. 7B and E), which indicated the immune suppression was lifted. As a result, the CTL effect was activated after treatment, reflected by the remarkable increase in the amount of CD8^+^ T cells (Fig. 7F). Of note, the reversal of TAM2 to TAM1 can increase CD8^+^ T cell infiltration.^39^ IFN-γ is a key cytokine secreted by CD8^+^ T cells for coordinating tumor immune responses and executing direct tumoricidal activity.^40^ It was found that with the treatment of the T12/Man-BSA NPs, an upregulating level of IFN-γ was determined in the glioma tissue (Fig. 7C).

It was expected that the DSF/Cu killed the cancer cells, and the dying cells disintegrated into immunogenic cell debris. In the presence of specific tumor antigens, macrophage-directed immunity can result in the stimulation of tumor-specific cytotoxic T cells.^34^ Therefore, the chemotherapy could work synergistically with the macrophage-induced immunotherapy.

**Fig. 8:**
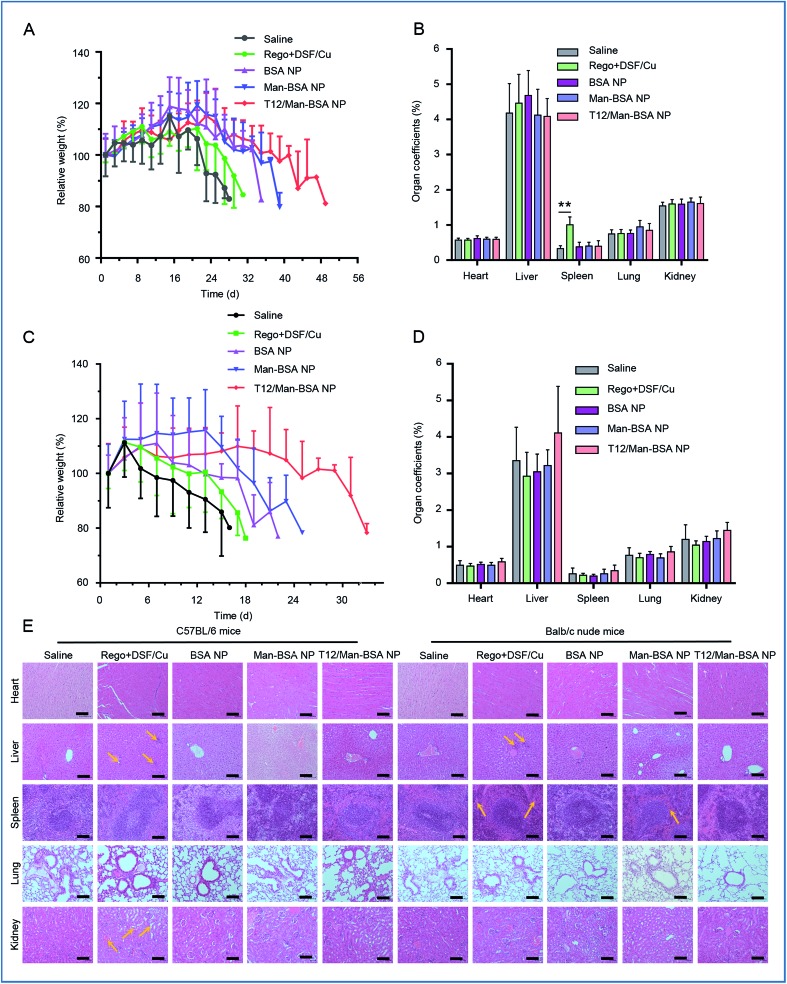
Biosafety evaluation. The body weight variations (A) and organ coefficients (B) in the Balbc/nude mice bearing U87 orthotopic glioma. The body weight variations (C) and organ coefficients (D) in the C57BL/6 mice bearing GL261 orthotopic glioma. (E) The histopathological examination of the major organs collected at the experimental endpoint (scale bar: 100 μm). The Rego + DSF/Cu treatment group pathologic symptoms specifically were included: hepatocellular focal necrosis; abnormal proliferation of the capillary and sinus expansion in the spleen; glomerular blood vessels and interstitial vasodilatation in the kidney.

The treatment biosafety was evaluated by monitoring the body weight change and measuring the organ coefficients of each group (Fig. 8A–D). Due to the high malignancy and aggressive progression of the orthotopic glioma model, all the groups showed body weight loss at the late stage of therapy. Compared with the saline control group, no significant difference in the organ coefficients was observed in the groups receiving the NPs. The histological examination showed that there were no obvious pathological changes in all the NP groups (Fig. 8E). This indicated the safety and biocompatibility of the proposed delivery system and treatment strategy.

## Discussion

Regorafenib is an oral multi-kinase inhibitor approved by the FDA, which can inhibit angiogenic kinases and the mutant oncogenic kinases KIT, RET, and B-RAF^41^,^42^ and modulate TAM re-polarization.^43^ DSF is a drug used for over six decades for preventing alcohol abuse. The potential and efficiency of DSF as an anticancer agent has been well demonstrated. In particular, a major anticancer mechanism is that DSF chelates the intratumoral Cu^2+^ and forms the DSF/Cu complex.^44^ The DSF/Cu can arrest the tumor growth *via* mechanisms of inhibition of cancer stem cells (CSCs) and proteasomes, and the induction of ROS production and apoptosis.^45^–^47^

A recent important finding, based on epidemiological analysis, reveals the connection between DSF and reduced cancer mortality and identifies the p97 segregase adaptor NPL4 as the anticancer target of DSF.^48^ The DSF-based therapy provides promise for cancer patients due to the ready availability of a low-cost drug with an already well-established PK, safety, and tolerance. Our study discovered that DSF also plays a role in glioma macrophage-directing immunotherapy, thus providing an attractive strategy for the clinical translation of an old drug for a new application. However, oral DSF was poorly taken up by the brain.^49^ For enhanced anti-glioma efficacy, a multifunctional biomimetic brain-targeting delivery strategy was developed in this work.

We demonstrated that the BBB endothelial cells overexpressed various nutrient transporters (*e.g.*, SPARC, TfRs, and GLUT-1) and MRs, and thus we proposed an albumin-based nanomedicine for biomimetic brain-targeting codelivery of DSF/Cu and Rego. The T12 peptide and mannose modified albumin nanoparticles can achieve multifunctional targeting delivery to the brain and penetrate the BBB. Meanwhile, SPARC and TfRs were also overexpressed in the glioma cells, and SPARC and MRs overexpressed in TAM2. Therefore, such a nanomedicine system can achieve “three-bird-one-stone” delivery.

The induction of clusters of albumin-binding proteins (ABP) on the cell surface is an essential step to activate enhanced albumin transport.^50^ Albumin NPs might act as a multivalent inducer for clustering the albumin-binding proteins because the NPs could bind with and cross-link multiple ABP receptors. In addition, it has been recently reported that intracellular delivery of the serum protein-based NPs was facilitated *via* micropinocytosis in Ras-activated glioma cells.^51^ Therefore, albumin-based nanomedicine provides a promising strategy for brain delivery.

MΦ as a potential drug target for neurological and psychiatric diseases has drawn great attention.^52^ Although MΦ has recently been explored as a drug carrier for brain cancer,^53^ its role in directing immunity against glioma has largely remained unknown. Our results demonstrated the reprograming MΦ increased the secretion of antitumor cytokines (*e.g.*, TNF-α and IFN-γ) and reduced the level of immune suppressor B7-H4. Importantly, it also took action on the T cells by inhibiting the Treg and activating the CD8^+^ T cells, and thus elicited strong cellular immunity.

## Conclusion

In summary, we developed a “two-birds-one-stone” biomimetic codelivery strategy for targeting the glioma microenvironment and macrophage-directed immunotherapy. BBB penetration can be achieved *via* biomimetic delivery pathways (*e.g.*, the nutrient transporters SPARC and TfRs). Meanwhile, SPARC and MRs overexpressed on the TAM2 can also serve as the delivery targets for the T12/Man-BSA NPs. An important benefit of such a treatment strategy is that the “re-education” of the pro-tumor TAM2 towards the antitumor TAM1 can lift the immune suppression and elicit CTL immune responses. The combination of immunotherapy and chemotherapy yielded enhanced treatment outcomes with reduced side toxicity.

## Experimental section

### Materials

Disulfiram (DSF), NHS-Cy5, and CFDA SE dye were obtained from Dalian Meilun Biotechnology Co., Ltd. (Dalian, China). Regorafenib (Rego) was purchased from Selleck Chemicals (USA). Copper(ii) chloride dihydrate was obtained from Sinopharm Chemical Reagent Co., Ltd. (Shanghai, China) and bovine serum albumin (BSA) from Amresco (USA). The derivative T12 peptide (sequence: CGGGTHRPPMWSPVWP) and T7 peptide (sequence: HAIYPRH) were synthesized by Bankpeptide biological technology co., Ltd (Hefei, China). Succinimidyl 4-(*N*-maleimidomethyl)cyclohexane-1-carboxylate (SMCC) was obtained from ProteoChem (Loves Park, USA) and 4-isothiocyanatophenyla-α-mannopyranoside from Chemsynlab Pharmaceutical Science & Technology Co., Ltd. (Beijing, China). Dulbecco’s modified Eagle’s Medium (DMEM) cell culture medium, fetal bovine serum (FBS), and 0.25% trypsin–EDTA were purchased from Gibco (USA). The Micro BCA protein assay kit was obtained from the Beyotime Institute of Biotechnology (Haimen, China). The 3-(4,5-dimethylthiazol-2-yl)-2,5-diphenyltetrazolium bromide (MTT), cocktail protease inhibitor, and lipopolysaccharide (LPS) were purchased from Sigma-Aldrich Co., Ltd (USA). Coumarin-6 was obtained from J&K Scientific Ltd (Shanghai, China). Murine M-CSF was obtained from Novus Biologicals (R&D system, USA). Murine IFN-γ and murine IL-4 were purchased from Peprotech (USA). The anti-transferrin receptor (TfR) antibody and anti-mannose receptor (CD206) antibody were from Abcam (UK). The anti-SPARC antibody was purchased from Cell Signaling Technology (USA). The anti-CD8 alpha antibody was obtained from Novus Biologicals (R&D system, USA). The anti-GLUT1 antibody, anti-B7-H4 antibody, and anti-foxp3 antibody were obtained from R&D Systems (USA). The mouse TNF-α and mouse TGF-β1 Elisa Kit were from Shanghai Dakewe Biotech Co., Ltd (Shanghai, China). The mouse IFN-γ Elisa Kit was purchased from R&D Systems (USA). SB203580 was from MedChemExpress (Monmouth Junction, USA). All other reagents were of analytical grade from Sinopharm Chemical Reagent Co., Ltd. (Shanghai, China).

### Cell lines

The U87 glioma cells genetically marked with a firefly luciferase reporter gene (U87-Luc) were kindly provided by Prof. J. Ding (Shanghai Institute of Materia Medica, Chinese Academy of Science). The murine glioma cell line GL261 was obtained from The Cell Bank of Chinese Academy of Sciences. Brain capillary endothelial cells (BCECs) were provided by Prof. J. X. Wang (School of Pharmacy, Fudan University).

### Animals

Balb/c nude mice (male, 4–5 weeks), C57BL/6 mice (male, 4–5 weeks), and Sprague Dawley rats (male, about 220 g) were supplied by the Shanghai Laboratory Animal Center (SLAC) Co., Ltd. (Shanghai, China), and housed at the SPF care facility with sterilized food pellets and distilled water under a 12 h light/dark cycle. All animal procedures were performed in accordance with the Guidelines for Care and Use of Laboratory Animals of Shanghai Institute of Materia Medica (SIMM), Chinese Academy of Sciences and approved by the Institutional Animal Care and Use Committee (IACUC) of SIMM. (Note: SIMM is an institution with AAALAC (The Association for Assessment and Accreditation of Laboratory Animal Care) International accreditation.)

## Methods

### Synthesis and characterization of the T12 peptide-modified BSA (T12-BSA)

SMCC (20 mg mL^–1^ in DMSO) was added dropwise to the BSA solution (20 mg mL^–1^ in PBS, pH 7.4) at a molar ratio of 3 : 1 and reacted under magnetic stirring for 1 h at room temperature. The activated BSA was purified by FPLC (ÄKTA purifier 10, GE Healthcare, USA) equipped with a desalting column (GE Healthcare, USA) to remove the excessive SMCC. The activated BSA was then mixed with Cys-GGG-T12 (sequence: CGGGTHRPPMWSPVWP) in PBS (pH 7.4) for 12 h at 4 °C. The T12-BSA conjugates were purified using a desalting column. The product content was determined using a standard BCA method and the product characterized using MALDI-TOF-MS.

### Synthesis and characterization of mannopyranoside-BSA (Man-BSA)

The 4-isothiocyanatophenyla-α-mannopyranoside (5 mg mL^–1^ in PBS, pH 7.4) was added dropwise to the BSA solution (20 mg mL^–1^ in PBS, pH 7.4) with a molar ratio of 3 : 1. The mixture was stirred for 4 h at room temperature and then purified using a desalting column to remove the excessive 4-isothiocyanatophenyla-α-mannopyranoside. The purified product content was determined using a standard BCA method and characterized using ESI-TOF.

### Preparation of drug-loaded albumin NPs

Albumin was denatured using the NaBH_4_ reduction-urea method from the previously described procedures.^16^ In brief, BSA, Man-BSA (with 50% BSA and 50% Man-BSA) or T12/Man-BSA (with 50% T12-BSA and 50% Man-BSA) was dissolved in 9.6 M urea solution (pH 8.3), followed by the addition of 0.1 mL of NaBH_4_ solution (75 mg mL^–1^, in 1 M NaOH solution) with 1.05 mL of ethanol as a defoamer. The mixture was incubated at room temperature, and then at 50 °C for 30 min, and lastly cooled to room temperature. DSF and CuCl_2_ ethanol solutions were mixed at a molar ratio of 1 : 1, and DSF chelated with Cu^2+^ (DSF/Cu) was thus formed. A total volume of 0.1 mL of DSF/Cu and Rego ethanol mixture was added dropwise to 0.5 mL of denatured albumin urea solution. Afterwards, 0.5 mL of ultra-pure water was added dropwise into the mixture under vortexing. The thus-formed drug-encapsulated NPs (defined as BSA NPs, Man-BSA NPs, and T12/Man-BSA NPs, respectively) were purified by a Sephadex G50 column (GE Healthcare, USA).

### Characterization of the three albumin NPs

The particle size of the NPs was measured using a dynamic light scattering instrument (Nano-ZS90, Malvern, UK). The morphology of the NPs was observed using transmission electron microscopy (TEM, Tecnai G2 Spirit, 120 kV) and atomic force microscopy (AFM, Bruker Dimension Icon, USA). The encapsulation efficiency and drug-loading capacity of the NPs were measured using HPLC (1260 Infinity, Agilent technologies, USA), and calculated by the following formulae: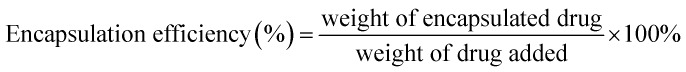 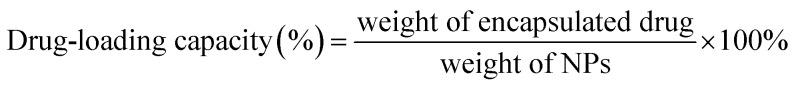


### Stability of the NPs and *in vitro* drug release

The three different albumin NPs were dispersed in the PBS (pH 7.4) containing 10% FBS to evaluate the stability by monitoring the change of the particle size at the different time points.

The *in vitro* drugs release from the NPs was conducted in PBS (pH 7.4) containing 0.5% w/v SDS with gentle shaking at 150 rpm at 37 °C using a dialysis membrane (MWCO 10–12 kDa). At the predefined time points, 0.5 mL of the dissolution medium was sampled and subjected to HPLC analysis, and an equivalent volume of fresh medium was replenished.

For DSF/Cu and Rego analysis, the mobile phase was Isocratic elution with a mixture of acetonitrile (85%, containing 0.1% trifluoroacetic acid) and ultra-pure water (15%, containing 0.1% trifluoroacetic acid) at a flow rate of 1.0 mL min^–1^ using a C_18_ column. The detection wavelength for DSF/Cu was 433 nm and Rego was 260 nm. The concentration was calculated using the predetermined calibration curves.

### Cell culture

The BCEC, GL261, and U87 cells were incubated in Dulbecco’s modified Eagle’s medium (DMEM, Gibco, USA) supplemented with 10% FBS and antibiotics (100 μg mL^–1^ of streptomycin and 100 U mL^–1^ of penicillin, Amresco, USA) in a humidified 5% CO_2_ incubator at 37 °C.

### Cellular uptake efficiency

The U87 cells were seeded in the 12-well plates at a density of 1 × 10^5^ cells per well. After incubation for 12 h, the cells were treated with the coumarin-6 loaded albumin NPs and incubated for 1 h. The cells were then thoroughly washed with PBS three times. The cellular uptake efficiency was determined by flow cytometry (FACS Calibur, Becton Dickinson, USA). In addition, the cells were fixed with 4% paraformaldehyde for 20 min and stained with DAPI for fluorescent imaging (fluorescence microscope, CARL ZEISS, Germany). Simultaneously, the U87 cell was pre-treated with SB203580 for 4 h then incubated with the NPs. The cellular uptake efficiency was determined by flow cytometry (FACS Calibur, Becton Dickinson, USA).

### Evaluation of the penetration ability across the *in vitro* BBB culture model

To establish the *in vitro* BBB model, the BCEC cells were seeded into the upper chambers of the Transwell cell culture plates (0.4 μm pore size, Corning, USA) at a density of 5 × 10^4^ cells per well. The integrity of the cultured monolayer model was tested by measuring the transendothelial electrical resistance (TEER, >200 Ω cm^2^) by an impedance instrument module (Millipore, USA). Then, the U87 cells were planted in the lower chambers at a density of 5 × 10^4^ cells per well 24 h before treatment. The coumarin-6-labeled NPs were added into the upper chambers and incubated at 37 °C for 4 h. The U87 cells at the lower chambers were collected and analyzed using flow cytometry (FACS Calibur, Becton Dickinson, USA). To investigate the penetration pathways, the BCEC cells at the upper chambers were pretreated with T12/T7 peptides and/or mannose (Man), and then incubated with the Man-BSA NPs or T12/Man-BSA NPs following the same procedures as described above.

### Penetration of the tumor spheroid

The U87 cells were seeded at a density of 2 × 10^3^ cells per well in a 96-well plate pretreated with 1% (w/v) agarose gel to prevent cell adhesion and cultured for 7 days. The tumor spheroids were incubated with the coumarin-6-labeled BSA NPs, Man-BSA NPs, or T12/Man-BSA NPs for 6 h (*n* = 3). The spheroids were then rinsed with PBS three times and subjected to confocal microscopy (TCS-SP8, Leica, Germany).

### Antiproliferative activity against glioma cells

The antitumor activity of DSF/Cu, Rego, the free combo drugs, and three different NPs were determined by a standard MTT assay of U87 and GL261 glioma cells. The cells were seeded in a 96-well plate at a density of 5 × 10^3^ cells per well. After 24 h, the cells were treated with various drugs (Rego, DSF/Cu, Rego + DSF/Cu, BSA NP, Man-BSA NP, or T12/Man-BSA NP) for 48 h. Subsequently, the MTT reagent (5 mg mL^–1^, 20 μL) was added to each well and incubated for another 4 h. After removal of the medium, 200 μL of DMSO was added to each well to dissolve the formazan crystals and the absorbance at 490 nm was measured by a microplate reader (Multiskan, Thermo Fisher, USA).

In addition, the U87 or GL261 cell apoptosis was investigated. The cells were seeded in a 6-well plate and cultured for 24 h. The cells were then incubated with the NPs (equal to DSF/Cu 0.6 μM and Rego 6 μM) or the free drugs for 24 h. After thorough washing with PBS, the cells were collected and stained with the FITC-Annexin V Apoptosis Detection Kit (Becton Dickinson, USA) according to the manufacturer’s protocol. The apoptotic cells were measured by flow cytometry (FACS Calibur, Becton Dickinson, USA).

### Mouse bone marrow-derived macrophage (BMM) culture and induced polarization

The bone marrow cells were collected from the C57BL/6 mice (male, 4–5 weeks, SPF) using a standard procedure. The bone marrow cells were rinsed with the serum-free DMEM medium and cultured in the fresh DMEM medium containing 30 ng mL^–1^ M-CSF for 48 h to induce BMM differentiation. IFN-γ and LPS were added to the medium for 24 h incubation to induce polarization towards the M1 phenotype and IL-4 for M2 polarization.

### *In vitro* M2 phenotype macrophage re-education study

The BMM-induced TAM1 or TAM2 culture medium was collected as the conditioned medium (BMM-CM). U87 cells were seeded at a density of 3 × 10^3^ cells per well in a 96-well plate and BMM-CM added for 24 h incubation. The cells were then exposed to various concentrations of DSF/Cu for 48 h and the cell viability was measured by a standard MTT assay.

TAM2 cellular uptake was also carried out. After BMM polarized into TAM2, the cells were incubated with NPs for 1 h and then thoroughly washed with PBS three times. The cellular uptake efficiency was determined by flow cytometry (FACS Calibur, Becton Dickinson, USA) and also fluorescent imaging (fluorescence microscope, CARL ZEISS, Germany).

A study on MΦ recruitment into tumor tissue from blood was examined. BMM (M1 polarization) were stained with CFDA SE for 30 min at 37 °C and then i.v. injected to the orthotopic U87 glioma-bearing mice with simultaneous co-administration with T12/Man-BSA NPs or subsequent administration with T12/Man-BSA NPs post 12 h. The saline injection was used as a control in parallel. After 12 h, the mice were sacrificed and the tumor tissues collected for confocal and FACS analysis.

In addition, TAM1 or TAM2 was treated with Rego or DSF/Cu or Rego + DSF/Cu for 48 h, and then the supernatant was collected to measure the cytokines using a standard ELISA method.

Furthermore, a co-culture model was developed with TAM1 or TAM2 in the upper chambers of the Transwell cell culture plate and U87 cells in the lower chambers. After co-culturing for 12 h, Rego (2 μM), DSF/Cu (0.2 μM), or Rego + DSF/Cu (2 + 0.2 μM) was added into both the upper and lower chambers and co-incubated for 24 h. The supernatant of TAM1 or TAM2 was collected for ELISA assay of the TNF-α and TGF-β1 levels according to the manufacturer’s protocol.

### The orthotopic glioma model

The orthotopic glioma model was developed by implanting the U87-Luc cells into the right striatum at a location 1.5 mm lateral to the bregma and at 3.5 mm using a stereotaxic apparatus. After surgery, the mice were maintained under the standard housing conditions for 10 days, and the orthotopic glioma was monitored using bioluminescence imaging performed in the IVIS imaging system (Caliper PerkinElmer, Hopkinton, MA, USA) by intraperitoneal injection of the luciferin substrate (150 mg kg^–1^, d-Luciferin Potassium Salt, PerkinElmer, USA).

### *In vivo* imaging

The U87 bearing mice were intravenously injected with Cy5-labeled albumin nanoparticles. The biodistribution of the NPs was monitored using an IVIS imaging system (Caliper PerkinElmer, Hopkinton, MA, USA) at the predetermined time points. At the end of the experiment, the mice were humanely sacrificed and the major organs (*e.g.*, heart, liver, spleen, lung, and kidney) and brain were collected and imaged to investigate the bio-distribution of the nanoparticles. The brains were then fixed with 4% paraformaldehyde for 48 h and then sucrose gradient dehydration for the preparation of cryosection slices (CM1950, Leica, Germany). The tissue sections were incubated overnight at 4 °C with anti-SPARC, anti-MR, or anti-TfR antibodies, and subsequently with an Alexa Fluor® 488-conjugated secondary antibody for 1 h, followed by DAPI staining. The tissue slices were imaged using confocal laser scanning microscopy (TCS-SP8, Leica, Germany).

### Pharmacokinetics study

The pharmacokinetics study was executed to compare the L-BSA NPs and T12/Man-BSA NPs. The Sprague Dawley rats were randomly divided into two groups (three rats per each group). The two NPs were injected by tail vein injection with the same dose (equal to 6 μg kg^–1^ Cy5 dye). The rats were anaesthetized and we collected 0.5 mL of blood by retro-orbital bleeding at predetermined time points. The plasma was obtained by centrifugation (5000 rpm, 10 min), and the concentration was measured by a fluorescence spectrophotometer (F-4600, HITACHI, Japan). The main PK parameters were fitted with DAS 2.0 PK software. Moreover, the bio-distribution of both NPs in the major organs was monitored by an IVIS imaging system (Caliper PerkinElmer, Hopkinton, MA, USA).

### *In vivo* anti-glioma treatment

The orthotopic U87 glioma-bearing mice were randomly divided into five groups (10 mice per group). The glioma growth was monitored using luciferin substrate for *in vivo* imaging. The mice were receiving saline (control), Rego + DSF/Cu (1.5 mg kg^–1^ each), and the albumin NPs (equal dose to the combo free drugs) *via* tail vein injection (q.o.d, five times). The survival rate and animal body weight change were recorded to evaluate the anti-glioma therapeutic efficacy. The animals were then humanely sacrificed, and the major organs (heart, liver, spleen, lung, and kidney) were collected and fixed at 48 h with 4% paraformaldehyde for histopathological examination to assess the adverse effects.

Meanwhile, the orthotopic GL261 glioma-bearing C57BL/6 mice were randomly divided into five groups (10 mice per group). After 7 days, the mice were receiving saline (control), Rego + DSF/Cu (1.5 mg kg^–1^ each), and the albumin NPs (equal dose to combo free drugs) *via* tail vein injection (q.o.d, five times). The survival rate and animal body weight change were recorded. The following procedures were consistent with the above-mentioned U87 glioma therapy.

### Mechanism studies

The U87 tumor tissues were dissected at the experimental endpoint and fixed with 4% paraformaldehyde for 48 h and then subjected to sucrose gradient dehydration for the preparation of the cryosection slices (CM1950, Leica, Germany). The tissue sections were stained M1 macrophages (CD80^+^) and M2 macrophages (CD206^+^), and subsequently had DAPI staining. The tissue slices were imaged using confocal laser scanning microscopy (TCS-SP8, Leica, Germany). The immunofluorescence of the CD8, B7-H4, and foxp3 expression in the GL261 tumor tissue was stained in parallel. The expression of the B7-H4 and MRs after treatment in the GL261 glioma tissues was also measured by western blotting. The IFN-γ level in the glioma tissues was detected using a standard ELISA method (ELISA kit for mouse IFN-γ, R&D Systems, Inc., USA).

### Statistical analyses

All data were analyzed by GraphPad Prism 6 software. The results were shown as mean ± SD (*n* > 3). The statistical analysis was performed by Student’s *t*-test and one-way ANOVA. The survival analysis was assessed using the Kaplan–Meier method by SPSS 16.0 software. The statistically significant difference was defined as *P < 0.05, **P < 0.01, and ***P < 0.001.

## Conflicts of interest

There are no conflicts to declare.

## Acronyms

AFM: Atomic force microscopy
BSA: Bovine serum albumin
BBB: Blood–brain barrier
BMM: Bone marrow-derived macrophage
CLSM: Confocal laser scanning microscopy
CTLs: Cytotoxic T lymphocytes
DSF/Cu: Disulfiram/copper complex
GLUT-1: Glucose transporters 1
IFN-α: Interferon α
MΦ: Macrophages
Man: Mannose
MR: Mannose receptor
NP: Nanoparticle
Rego: Regorafenib
SPARC: Secreted protein acidic and rich in cysteine
TAM: Tumor-associated macrophage
TAM1: M1 phenotype TAM
TAM2: M2 phenotype TAM
TEM: Transmission electron microscopy
TGF-β: Transforming growth factor β
TNF-α: Tumor necrosis factor alpha
TME: Tumor microenvironment
TMZ: Temozolomide
TfR: Transferrin receptor
Treg: Regulatory T cells
T12: Transferrin receptor-binding peptide 12

## Supplementary Material

Supplementary information
