# Supplementary material for: Dual-targeting biomimetic delivery for anti-glioma activity via remodeling the tumor microenvironment and directing macrophage-mediated immunotherapy
Source: Chem Sci. 2018 Jan 31;9(10):2674–89. doi: 10.1039/c7sc04853j (PMC5914428; doi:10.1039/c7sc04853j)

Electronic Supplementary Material (ESI) for Chemical Science.
This journal is © The Royal Society of Chemistry 2018

Supporting Information

Dual-Targeting Biomimetic Delivery for Anti-glioma via Remodeling Tumor Microenvironment and Directing Macrophage-Mediated Immunotherapy

*Pengfei Zhao [a, b, †], Yonghui Wang [a, †], Xuejia Kang [a, d], Aihua Wu [a, d], Weimin Yin [a, c], Yisi Tang [a, d], Jinyu Wang [a], Meng Zhang [a], Yifei Duan [c], Yongzhuo Huang *[a]*

Table S1 Characterization of the NPs.

	BSA NP	BSA/Man-BSA NP	T12-BSA/Man-BSA NP
Particle mean size (nm)	123.7 ± 2.7	128.0 ± 1.2	132.2 ± 2.1
PDI	0.11 ± 0.019	0.087 ± 0.017	0.14 ± 0.011
Zeta potential (mV)	-16.8 ± 0.31	-17.1 ± 0.56	-18.7 ± 0.15
Encapsulation efficiency (Rego, %)	50.41 ± 1.37	49.73 ± 1.53	46.54 ± 1.04
Encapsulation efficiency (DSF/Cu, %)	74.75 ± 2.34	80.43 ± 2.01	83.32 ± 1.55
Drug loading (Rego, %)	1.48 ± 0.22	1.60 ± 0.17	1.55 ± 0.34
Drug loading (DSF/Cu, %)	2.35 ± 0.19	2.44 ± 0.15	2.49 ± 0.21

Table S2 Statistical analysis of U87 orthotopic glioma survival curve

	Saline		Rego+DSF/Cu		BSA NP		Man-BSA NP		T12/Man-BSA NP	
	Chi-Square	Sig.	Chi-Square	Sig.	Chi-Square	Sig.	Chi-Square	Sig.	Chi-Square	Sig.
Saline			2.940	0.086	7.369	0.007	19.185	0.0001	21.461	0.0001
Rego+DSF/Cu	2.940	0.086			2.813	0.094	13.301	0.0001	21.873	0.0001
BSA NP	7.369	0.007	2.813	0.094			4.793	0.029	17.479	0.0001
Man-BSA NP	19.185	0.0001	13.301	0.0001	4.793	0.029			10.800	0.001
T12/Man-BSA NP	21.461	0.0001	21.873	0.0001	17.479	0.0001	10.800	0.001		

Table S3 Statistical analysis of GL261 orthotopic glioma survival curve

	Saline		Rego+DSF/Cu		BSA NP		Man-BSA NP		T12/Man-BSA NP	
	Chi-Square	Sig.	Chi-Square	Sig.	Chi-Square	Sig.	Chi-Square	Sig.	Chi-Square	Sig.
Saline			4.875	0.027	11.993	0.001	20.085	0.0001	21.332	0.0001
Rego+DSF/Cu	4.875	0.027			5.947	0.015	14.607	0.0001	18.963	0.0001
BSA NP	11.993	0.001	5.947	0.015			3.020	0.082	12.884	0.0001
Man-BSA NP	20.085	0.0001	14.607	0.0001	3.020	0.082			5.609	0.018
T12/Man-BSA NP	21.332	0.0001	18.963	0.0001	12.884	0.0001	5.609	0.018		

Figure S1 Cytotoxicity test in U87 cells (A) and GL261 cells (C). The apoptosis assay in U87 cells (B) and GL261 cells (D).

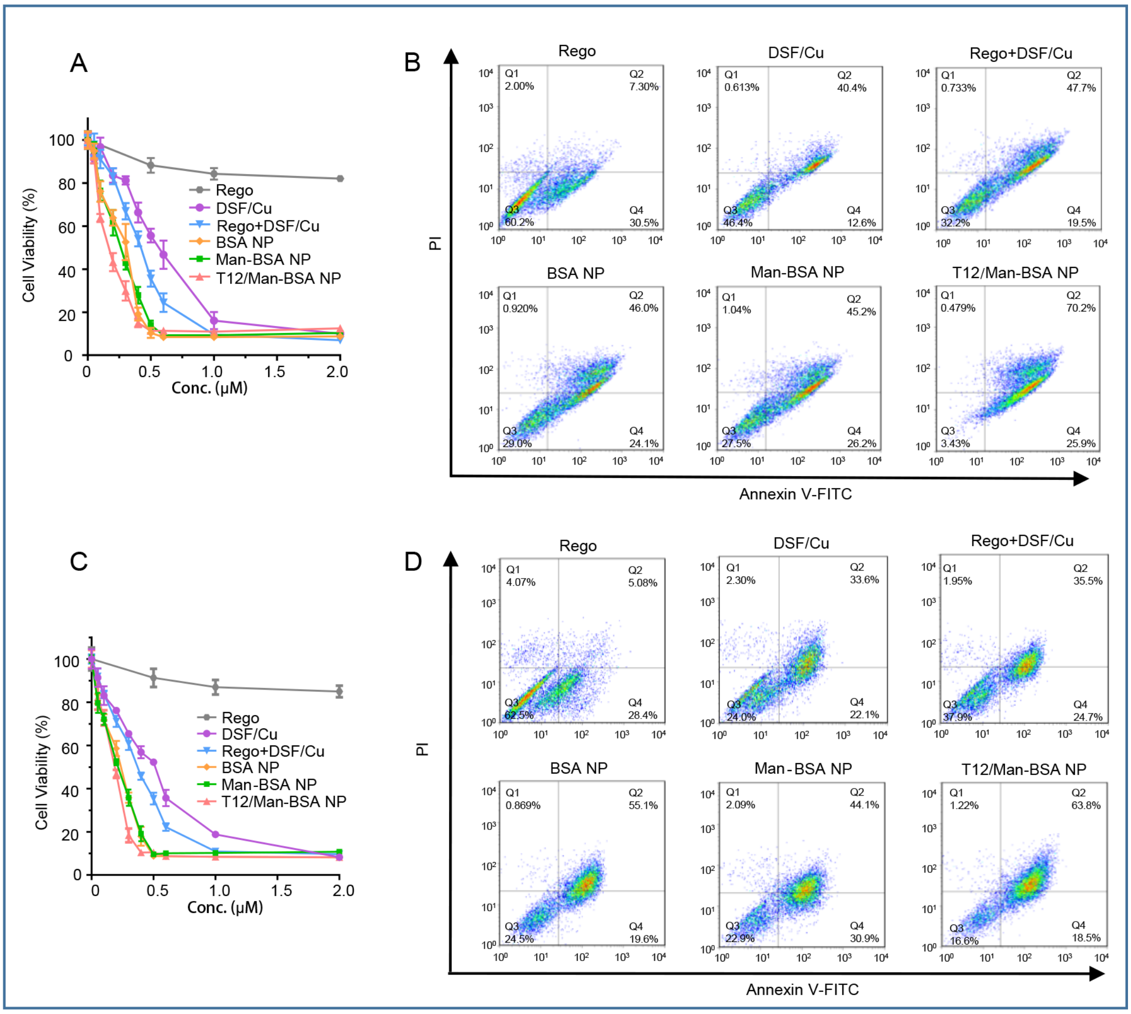

Figure S2 (A) ESI-TOF-MS of Man-BSA. (B) MALDI-TOF-MS of T12-BSA.

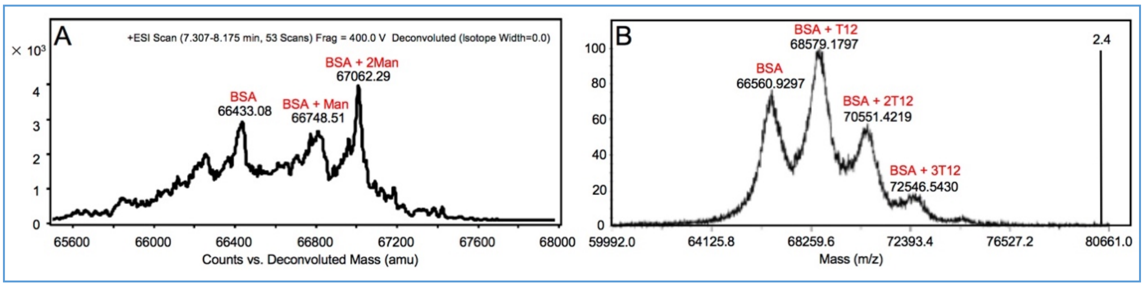

Figure S3 (A) Fluorescence images of the cellular uptake of the NPs in TAM2 (scale bar: 50 μm). (B) FACS analysis of cellular uptake efficiency of the NPs in TAM2 cells. (C) FACS analysis of the cellular uptake efficiency of the NPs in U87 cells. (D) FACS analysis of uptake efficiency of the NPs in U87 cells after incubation with the p38-MAPK inhibitor SB203580 (0, 10, or 50 μM). (E) Effects of SB203580 on SPARC expression in U87 cells (0, 1, 10, or 50 μM).

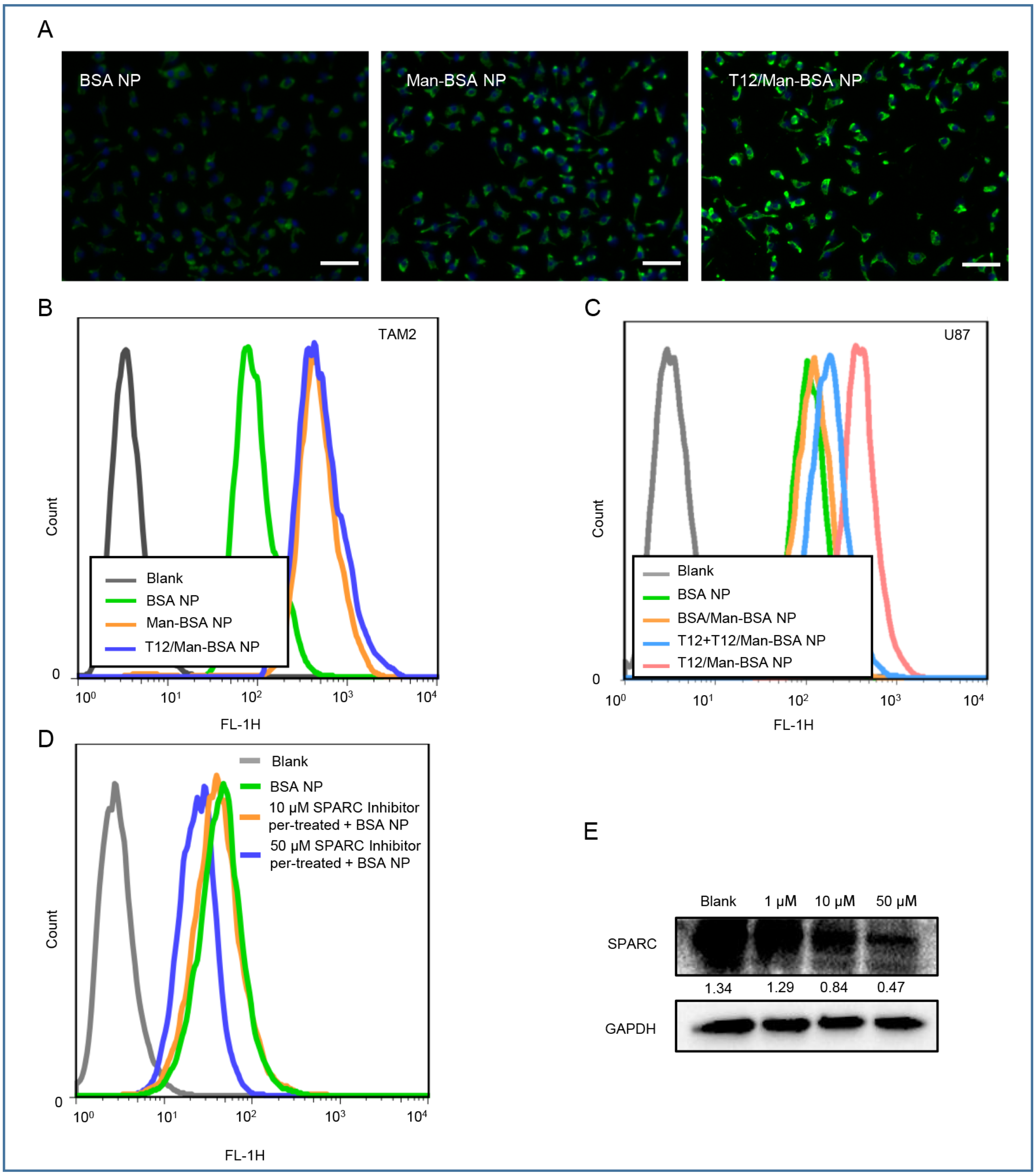

Figure S4 (A) Pharmacokinetics curves and main parameters of the L-BSA NPs and T12/Man-BSA NPs. (B) Imaging of major organs dissected from the mice of the L-BSA NPs and T12/Man-BSA NPs groups. (C) The radiant efficiency of the organs.

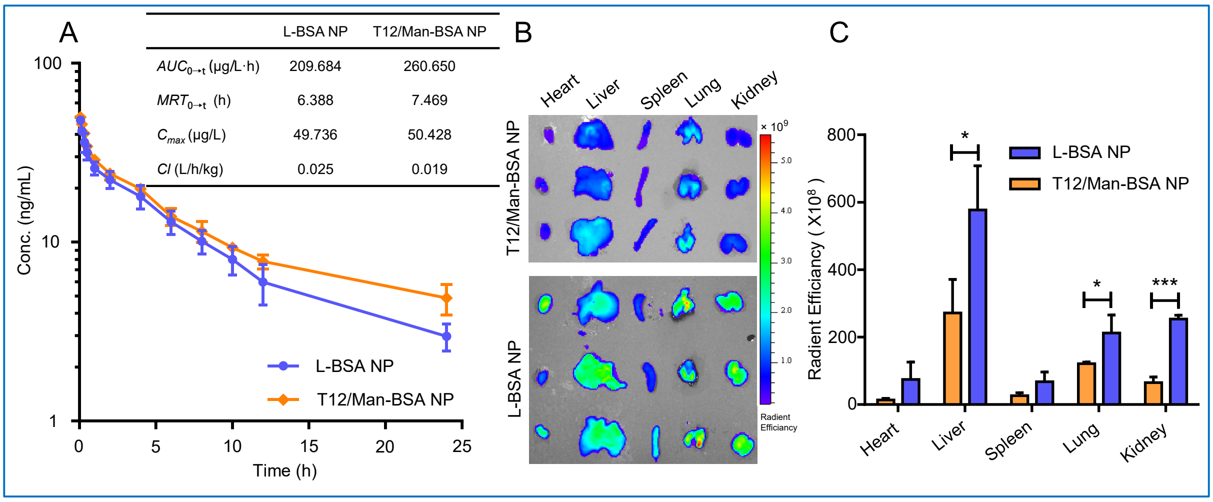

Figure S5 Immunofluorescence colocalization of the Cy5-Man-BSA NPs and GLUT-1.

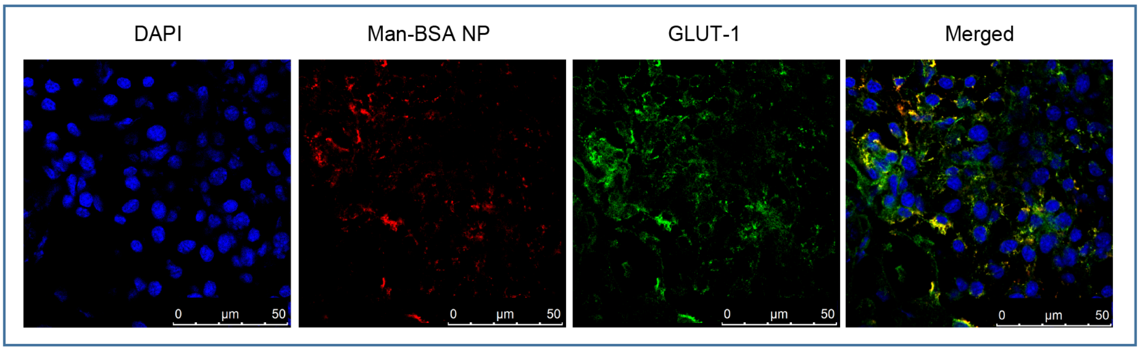

Figure S6 Study of MΦ recruited from blood into tumor tissue. BMM (M1 polarization) were stained with CFDA SE and injected to the tumor-bearing mice by tail vein. The tumor tissues were collected for confocal and FACS analysis after 12 h.

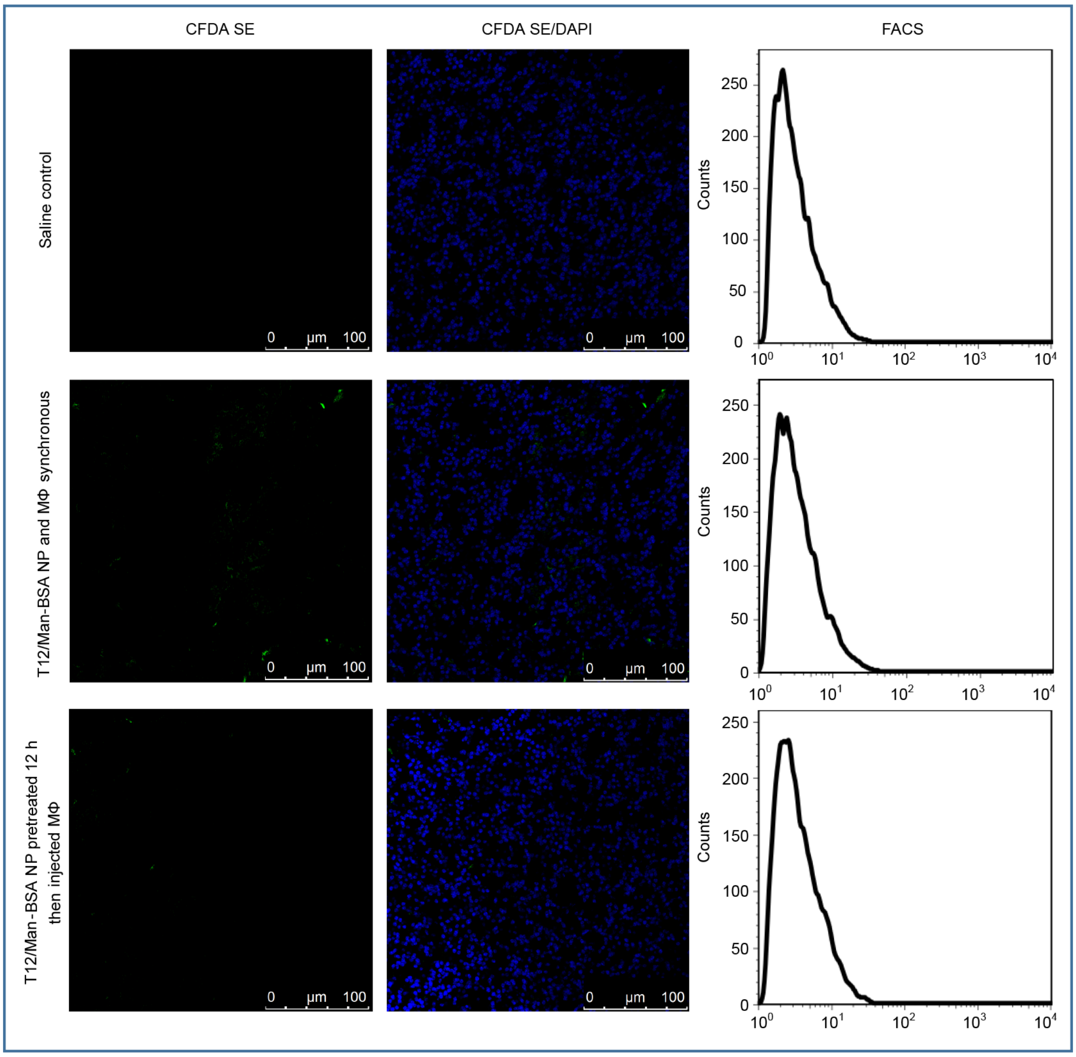

Supplement: Supplementary file 1 [file SC-009-C7SC04853J-s001.pdf]
